# Gastric perforation from closed-loop obstruction created by a gastric band and distal small bowel obstruction: a case report

**DOI:** 10.1093/jscr/rjag594

**Published:** 2026-07-16

**Authors:** Gregory Kunis, John Pollock, Erin Hart, Alexandra Ford, Stephanie Giorno, Rebecca Schmehl, Mingwei Ni

**Affiliations:** General Surgery, Christiana Care Hospital, 4755 Ogletown Stanton Rd, Newark, DE 19718, United States; General Surgery, Christiana Care Hospital, 4755 Ogletown Stanton Rd, Newark, DE 19718, United States; General Surgery, Christiana Care Hospital, 4755 Ogletown Stanton Rd, Newark, DE 19718, United States; General Surgery, Christiana Care Hospital, 4755 Ogletown Stanton Rd, Newark, DE 19718, United States; General Surgery, Christiana Care Hospital, 4755 Ogletown Stanton Rd, Newark, DE 19718, United States; General Surgery, Christiana Care Hospital, 4755 Ogletown Stanton Rd, Newark, DE 19718, United States; General Surgery, Christiana Care Hospital, 4755 Ogletown Stanton Rd, Newark, DE 19718, United States

**Keywords:** gastric banding, obstruction, perforation, closed-loop

## Abstract

Complications secondary to laparoscopic adjustable gastric banding are well defined in the literature, and this procedure has fallen out of favor due to more effective weight-loss surgeries over the last 15 years. Small bowel obstruction (SBO) due to gastric band erosion, slippage, compression/adhesion from the silicone tube, and bowel incarceration between abdominal wall and silicone tube are well documented in the literature. This case demonstrates a patient with an adhesive SBO related to a prior nephrectomy, who also had a gastric band in place, leading to a closed-loop obstruction. The closed-loop obstruction in this case was caused from the gastric band proximally and an adhesion distally, culminating in a gastric perforation. This complication of perforation is not well described in the literature and should be brought to the attention of our surgical community as a potential complication of patients with gastric bands and the potential for adhesive SBOs.

## Introduction

Background: In the mid-2000s, laparoscopic adjustable gastric bands (LAGB) accounted for almost 50% of all bariatric surgeries. However, in the last 10 years this number has dropped to <1% [1]. Gastric bands ended up with high revision and removal rates and as more advanced and efficacious weight loss surgeries and medications arrived, the bands were put to the side. About 50% of gastric bands require revision, including ~25% for major late complications such as slippage, prolapse, pouch dilation, gastric reflux, port/tubing malfunction, band erosion, and obstruction [2]. Of these complications, band erosion and obstruction are two of the most severe complications. A 2024 narrative review by Vitiello *et al*. [3] found 43 case reports of intestinal obstruction after LAGB, emphasizing the rarity of this complication. Most of these obstructions were caused by either band migration and erosion or internal hernia due to a loose tube loop. Band erosion is also rare but is seen more frequently with some studies citing anywhere from 1%–10% of major complications as erosions [4].

As noted above, small bowel obstructions (SBO) are not seen very commonly as complications of LAGB. However, they are one of the most common surgical emergencies generally, accounting for ~15% of all hospital admissions for acute abdominal pain and ~ 20% of all cases of acute surgical abdomen in adults [5]. In the United States, SBO results in ~300 000 hospital admissions each year [5]. The mechanism and causes of SBO are vast, but most (~60%–75%) of all SBOs in the US occur due to intra-abdominal adhesive disease secondary to prior abdominal or pelvic surgery [6].

The key teaching point of this case is the interaction between two obstructive processes. In patients without a gastric band, vomiting or nasogastric decompression may partially decompress the stomach and proximal small bowel. In patients with a functionally restrictive LAGB, however, proximal decompression may be impaired at the gastroesophageal junction (GEJ). When a second distal transition point develops from adhesive SBO, the stomach and proximal bowel may become trapped between a proximal restrictive point and a distal obstructive point, creating a closed-loop physiology with rapid gastric distension. Campbell *et al*. previously reported closed-loop obstruction in patients with LAGB and SBO, and Drober *et al*. recently described closed-loop obstruction between a gastric band and an obstructed ventral hernia [6, 7]. The present case differs in that the closed-loop physiology progressed to gastric pneumatosis, ischemia, and fundal perforation.

## Case report

The patient was a 62-year-old male with a past medical history of renal cell carcinoma status post right nephrectomy and obesity status post LAGB who presented to the emergency department with acute-onset abdominal pain, nausea, and vomiting. He was found to be tachycardic with a normal blood pressure, and labs were notable for significant leukocytosis (white blood cells = 20.0 × 10e^3^μL). He had a computed tomography (CT) scan of his abdomen and pelvis which demonstrated his gastric band in place. However, severe distension of the stomach with pneumatosis of the posterior-lateral wall of the gastric cardia and fundus, extraluminal gas in the anterior abdomen, free fluid in the left upper quadrant, and dilation of the small bowel with a transition point in the right lower quadrant (RLQ) were also seen (Figs 1–4).

**Figure 1:**
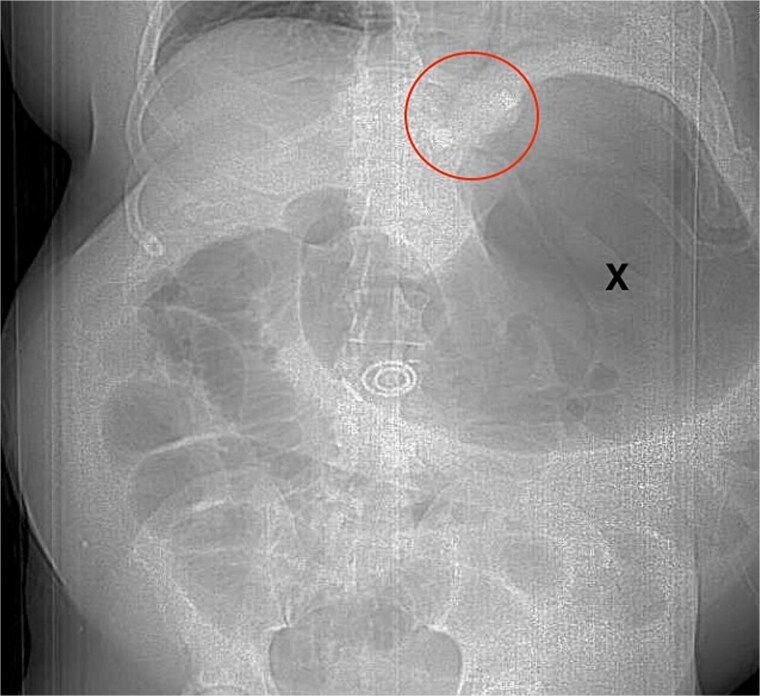
Scout film X-ray showing closed-loop obstruction in the patient; circle shows gastric band; x shows distended stomach.

**Figure 2:**
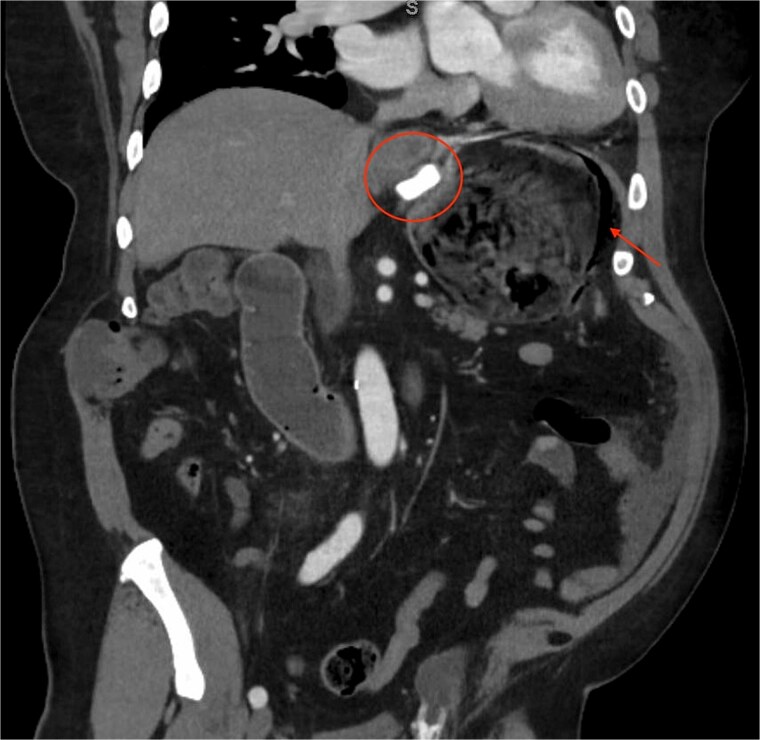
Coronal CT showing gastric band, distension, pneumatosis; circle shows gastric band, arrow pointing to pneumatosis in the stomach.

**Figure 3:**
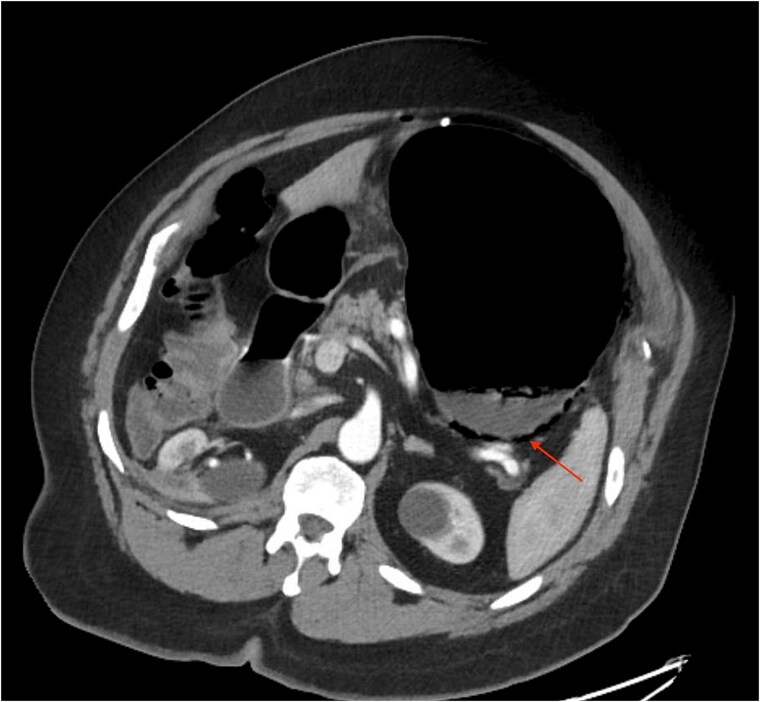
Axial CT showing gastric distension and pneumatosis; arrow showing pneumatosis of the fundus and area of suspected perforation.

**Figure 4:**
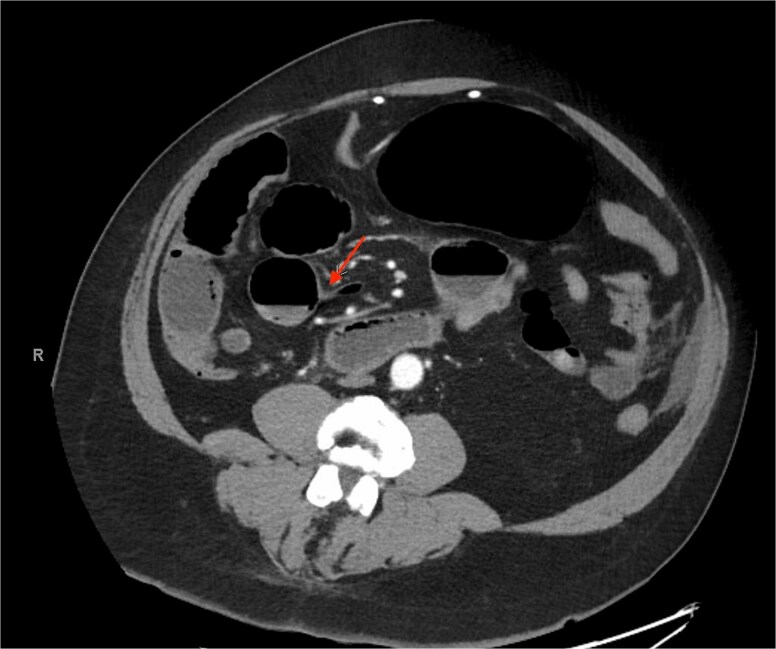
Axial CT showing small bowel transition point; arrow showing distal adhesive transition point, which completes the closed-loop system together with the gastric band.

Due to the concern for bowel perforation, the patient was taken urgently to the operating room. The procedure was started in a laparoscopic fashion, as the patient was stable, and we regularly perform acute care surgery cases in a minimally invasive fashion at our institution when safe to do so, to save the patient the morbidity of an exploratory laparotomy. On initial examination, the stomach was found to be severely distended, with bilious ascites in the abdomen, consistent with a gastric perforation. The gastric wrap was taken down, pseudo-capsule over the lap band was dissected, and the band was unbuckled and removed. Next, the gastric fundus was examined which showed patchy ischemia and a small 1 cm perforation. The stomach was decompressed using an endoscope and perfusion returned to the area of concern. The perforation was small enough for primary repair, which was accomplished using 3–0 absorbable V-loc suture in a two-layer fashion. Finally, attention was turned to the distal point of obstruction in the small bowel. Unfortunately, the adhesions causing the distal point of obstruction were unable to be lysed laparoscopically, so the approach was converted to laparotomy. The adhesions were identified in the RLQ and lysed, and the obstruction resolved with no need for bowel resection. The patient recovered well, was advanced to a full liquid diet, and was discharged 7 days later.

## Discussion

In this case, a patient with previous abdominal surgery (nephrectomy) presented with a SBO secondary to adhesive disease deep in his abdomen. The proposed mechanism is a two-point obstruction. Proximally, the LAGB can act as a fixed or functional narrowing at the GEJ. This may limit emesis and may also make decompression via nasogastric tube incomplete or technically difficult. Distally, an adhesive SBO created a second transition point. As time passes, fluid, swallowed air, gastric secretions, and intestinal contents accumulate between these two points, intraluminal pressure rises rapidly, and the stomach becomes part of a closed-loop system. This acute massive gastric dilation (AMGD) can cause ischemic necrosis and perforation due to the pressure on the gastric wall exceeding the perfusion capability. This physiology is commonly seen along the fundus or greater curvature regions and is most commonly associated with eating disorders, post-abdominal surgery, SBOs, pyloric stenosis, duodenal obstructions, and acute psychogenic polydipsia [8]. The fundus is particularly vulnerable to this physiology, as it can become the most distended portion of the stomach impairing venous outflow before arterial inflow, producing congestion, ischemia, pneumatosis, necrosis, and eventual perforation.

The clinical implication of this case is that standard adhesive SBO treatment algorithms may require modification when an intact gastric band is present. In an otherwise stable patient with adhesive SBO and no signs of strangulation, ischemia, or peritonitis, nonoperative management with bowel rest, intravenous fluids, and decompression is often appropriate [4, 5]. However, in a patient with LAGB, the band may prevent the usual protective pathway of proximal decompression. Initial management should include early surgical consultation, prompt CT evaluation, aggressive resuscitation, and consideration of immediate band deflation if an adjustable port is accessible. However, severe gastric dilation, inability to pass or effectively decompress with a nasogastric tube, persistent retching without emesis, gastric pneumatosis, free air, peritonitis, sepsis, rising lactate, or failure of early decompression should lower the threshold for urgent operative exploration.

## Conclusion

Closed-loop obstruction has been reported in patients with gastric bands, but this case highlights a severe and underrecognized consequence: distal adhesive SBO combined with proximal LAGB restriction can progress to AMGD, fundal ischemia, and perforation. In patients with LAGB who present with SBO physiology, early recognition, early decompression or band deflation when feasible, and a lower threshold for operative intervention are essential when gastric ischemia, perforation, or failed decompression is suspected.
